# Algorithmic Analysis of Blockchain Efficiency with Communication Delay

**DOI:** 10.1007/978-3-030-45234-6_20

**Published:** 2020-03-13

**Authors:** Carlos Pinzón, Camilo Rocha, Jorge Finke

**Affiliations:** 8 University of Paderborn, Paderborn, Germany; 9 ICREA, Open University of Catalonia, Barcelona, Spain; Pontificia Universidad Javeriana, Cali, Colombia

## Abstract

A blockchain is a distributed hierarchical data structure. Widely-used applications of blockchain include digital currencies such as Bitcoin and Ethereum. This paper proposes an algorithmic approach to analyze the efficiency of a blockchain as a function of the number of blocks and the average synchronization delay. The proposed algorithms consider a random network model that characterizes the growth of a tree of blocks by adhering to a standard protocol. The model is parametric on two probability distribution functions governing block production and communication delay. Both distributions determine the synchronization efficiency of the distributed copies of the blockchain among the so- called workers and, therefore, are key for capturing the overall stochastic growth. Moreover, the algorithms consider scenarios with a fixed or an unbounded number of workers in the network. The main result illustrates how the algorithms can be used to evaluate different types of blockchain designs, e.g., systems in which the average time of block production can match the average time of message broadcasting required for synchronization. In particular, this algorithmic approach provides insight into efficiency criteria for identifying conditions under which increasing block production has a negative impact on the stability of a blockchain. The model and algorithms are agnostic of the blockchain’s final use, and they serve as a formal framework for specifying and analyzing a variety of non-functional properties of current and future blockchains.

## 1 Introduction

A blockchain is a distributed hierarchical data structure that cannot be modified (retroactively) without alteration of all subsequent blocks and the consensus of a majority. It was invented to serve as the public transaction ledger of Bitcoin [22]. Instead relying on a trusted third party, this digital currency is based on the concept of 'proof-of-work', which allows users to execute payments by signing transactions using hashes through a distributed time-stamping service. Resistance to modifications, decentralized consensus, and robustness for supporting cryptocurrency transactions, unleashes the potential of blockchain technology for uses in various industries, including financial services [12,26,3], distributed data models [5], markets [25], government systems [15,23], healthcare [13,1,18], IoT [16], and video games [21].

Technically, a blockchain is a distributed append-only data structure comprising a linear collection of blocks, shared among so-called *workers*, also referred often as *miners*. These miners generally represent computational nodes responsible for working on extending the blockchain with new blocks. Since the blockchain is decentralized, each worker possesses a local copy of the blockchain, meaning that two workers can build blocks at the same time on unsynchronized local copies of the blockchain. In the typical peer-to-peer network implementation of blockchain systems, workers adhere to a consensus protocol for inter-node communication and validation of new blocks. Specifically, workers build on top of the largest blockchain. If they encounter two blockchains of equal length, then workers select the chain whose last produced block was first observed. This protocol generally guarantees an effective synchronization mechanism, provided that the task of producing new blocks is hard to achieve in comparison to the time it takes for inter-node communication. The effort of producing a block relative to that of communicating among nodes is known in the literature as 'proof of work'. If several workers extend different versions of the blockchain, the consensus mechanism enables the network to eventually select only one of them, while the others are discarded (including the data they carry) when local copies are synchronized. The synchronization process persistently carries on upon the creation of new blocks.

The scenario of discarding blocks massively, which can be seen as an efficiency issue in a blockchain implementation, is rarely present in "slow" block-producing blockchains. The reason is that the time it takes to produce a new block is long enough for workers to synchronize their local copies of the blockchain. Slow blockchain systems avert workers from wasting resources and time in producing blocks that are likely to be discarded in an upcoming synchronization. In Bitcoin, for example, it takes on average 10 minutes for a block to be produced and only 12.6 seconds to communicate an update [8]. The theoretical fork-rate of Bitcoin in 2013 was approximately 1.78% [8]. However, as the blockchain technology finds new uses, it is being argued that block production needs to be faster [6,7]. Broadly speaking, understanding how speed-ups in block production can negatively impact blockchains, in terms of the number of blocks discarded due to race conditions among the workers, is important for designing new fast and yet efficient blockchains.

This paper introduces a framework to formally study blockchains as a particular class of random networks with emphasis in two key aspects: the speed of block production and the network synchronization delays. As such, it is parametric on the number of workers under consideration (possibly infinite), the probability distribution function that specifies the time for producing new blocks, and the probability distribution function that specifies the communication delay between any pair of randomly selected workers. The model is equipped with probabilistic algorithms to simulate and formally analyze blockchains concurrently producing blocks over a network with varying communication delays. These algorithms focus on the analysis of the continuous process of block production in *fast* and highly distributed systems, in which inter-node communication delays are crucial. The framework enables the study of scenarios with fast block production, in which blocks tend to be discarded at a high rate. In particular, it captures the trade-off between speed and efficiency. Experiments are presented to understand how this trade-off can be analyzed for different scenarios. As fast blockchain systems tend to spread to novel applications, the algorithmic approach provides mathematical tools for specifying, simulating, and analyzing blockchain systems.

It is important to highlight that the proposed model and algorithms are agnostic of the concrete implementation and final use of the blockchain system. For instance, the 'rewards' for mining blocks such as the ones present in the Bitcoin network are not part of the model and are not considered in the analysis algorithms. On the one hand, this sort of features can be seen as particular mechanisms of a blockchain implementation that are not explicitly required for the system to evolve as a blockchain. Thus, including them as part of the framework can narrow its intended aim as a general specification, design, and analysis tool. On the other hand, such features may be abstracted away into the proposed model by tuning the probability distribution functions that are parameters of the model, or by considering a more refined base of choices among the many probability distribution functions at hand for a specific analysis. Therefore, the proposed model and algorithms are general enough to encompass a wide variety of blockchain systems and their analysis.

The contribution of this work is threefold. First, a random network model is introduced (in the spirit of, e.g., Barabasi-Albert [4] and Erdös-Renyi [9]) for specifying blockchains in terms of the speed of block production and communication delays for synchronization among workers. Second, exact and approximation algorithms for the analysis of blockchain efficiency are made available. Third, based on the proposed model and algorithms, empirical observations about the tensions between production speed and synchronization delay are provided.

The remaining sections of the paper are organized as follows. Section 2 summarizes basic notions of proof-of-work blockchains. Sections 3 and 4 introduce the proposed network model and algorithms. Section 5 presents experimental results on the analysis of fast blockchains. Section 6 relates these results to existing research, and draws some concluding remarks and future research directions.

## 2 An Overview of Proof-of-work Blockchains

This section overviews the concept of proof-of-work distributed blockchain systems and introduces basic definitions, which are illustrated with the help of an example.

A *blockchain* is a distributed hierarchical data structure of blocks that cannot be modified (retroactively) without alteration of all subsequent blocks and the consensus of the network majority. The nodes in the network, called *workers*, use their computational power to generate *blocks* with the goal of extending the blockchain. The adjective 'proof-of-work' comes from the fact that producing a single block for the blockchain tends to be a computationally hard task for the workers, e.g., a partial hash inversion.

**Definition 1.** *A* block *is a digital document containing: (i) a digital signature of the worker who produced it; (ii) an easy to verify proof-of-work witness in the form of a nonce; and (iii) a hash pointer to the previous block in the sequence (except for the first block, called the* origin*, that has no previous block and is unique).*

Technical definitions of blockchain as a data structure have been proposed by different authors (see, e.g., [27]). Most of them coincide on it being an immutable, transparent, and decentralized data structure shared by all workers in the network. For the purpose of this paper, it is important to distinguish between the *local* copy, independently owned by each worker, and the abstract *global* blockchain, shared by all workers. The latter holds the complete history of the blockchain.

**Definition 2.** *The* local blockchain *of a worker $w$ is a non-empty sequence of blocks stored in the local memory of $w$. The* global blockchain *(or,* blockchain*) is the minimal rooted tree containing all workers' local blockchains as branches.*

Under the assumption that the origin is unique (Definition 1), the (global) blockchain is well-defined for any number of workers present in the network. If there is at least one worker, then the blockchain is non-empty. Definition 2 allows for local blockchains to be either synchronized or unsynchronized. The latter is common in systems with long communication delays or in the presence of anomalous situations (e.g., if a malicious group of workers is holding a fork intentionally). As a consequence, the global blockchain cannot simply be defined as a unique sequence of blocks, but rather as a distributed data structure against which workers are assumed to be partly synchronized to.

Figure 1 presents an example of a blockchain with five workers, where blocks are represented by natural numbers. On the left, the local blockchains are depicted as linked lists; on the right, the corresponding global blockchain is depicted as a rooted tree. Some of the blocks in the rooted tree representation in Figure 1 are labeled with the identifier of a worker, which indicates the position of each worker in the global blockchain. For modeling, the rooted tree representation of a blockchain is preferred. On the one hand, it can reduce the amount of memory needed for storage and, on the other hand, it visually simplifies the inspection of the data structure. Furthermore, storing a global blockchain with $m$ workers containing $n$ unique blocks as a collection of lists requires in the worst-case scenario $O(mn)$ memory (i.e., with perfect synchronization). In contrast, the rooted tree representation of the same blockchain with $m$ workers and $n$ unique blocks requires $O(n)$ memory for the rooted tree (e.g., using parent pointers) and an $O(m)$ map for assigning each worker its position in the tree, totaling $O(n + m)$ memory.

A blockchain tends to achieve synchronization among the workers due to the following reasons. First, workers follow a *standard protocol* in which they are constantly trying to produce new blocks and broadcasting their achievements to the entire network. In the case of cryptocurrencies, for instance, this behavior is motivated by paying a reward. Second, workers can easily verify (i.e., with

$$
\begin{aligned}
w_0 &: 0 \prec 1 \prec 5 \\
w_1 &: 0 \prec 2 \prec 3 \prec 6 \\
w_2 &: 0 \prec 2 \prec 4 \\
w_3 &: 0 \prec 2 \prec 3 \\
w_4 &: 0 \prec 2 \prec 3 \prec 6
\end{aligned}
$$

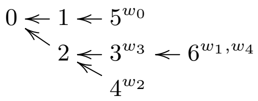

Fig. 1: A blockchain network of five workers with their local blockchains (left) and the corresponding global blockchain (right); blocks are represented by natural numbers. Workers $w_0$, $w_2$, and $w_3$ are not yet synchronized with the longest sequence of blocks.

a fast algorithm) the authenticity of any block. If a malicious worker (i.e., an *attacker*) changes the information of one block, that worker is forced to repeat the extensive proof-of-work process for that block and all its subsequent blocks in the blockchain. Otherwise, its malicious modification cannot become part of the global blockchain. Since repeating the proof-of-work process requires that the attacker spends a prohibitively high amount of resources (e.g., electricity, time, and/or machine rental), such a situation is unlikely to occur. Third, the standard protocol forces any malicious worker to confront the computational power of the whole network, assumed to have mostly honest nodes.

Algorithm 1 presents a definition of the above-mentioned standard protocol, which is followed by each worker in the network. When a worker produces a new block, it is appended to the block it is standing on, moves to it, and notifies the network about its current position and new distance to the root. Upon reception of a notification, a worker compares its current distance to the root with the incoming position. Such a worker switches to the incoming position whenever it represents a greater distance. To illustrate the use of the standard protocol with a simple example, consider the blockchains depicted in figures 1 and 2. In the former, either $w_1$ or $w_4$ produced block 6, but the other workers are not yet aware of its existence. In the latter, most of the workers are synchronized with the longest branch, which is typical of a slow blockchain system, and results in a tree with few and short branches.

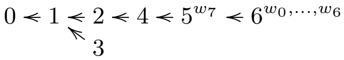

Fig. 2: Example of a typical slow system with few and short branches.

Some final remarks on inter-node communication and implementations for enforcing the standard protocol are due. Note that message communication in the standard protocol is required to include enough information about the position of a worker to be located in the tree. The detail degree of this information depends, generally, on the design of the particular blockchain system. On the one hand,

**Algorithm 1:** Standard protocol for each worker $w_i$ in a blockchain.

```
B_i ← [origin]
do forever
    do in parallel, stop on first to occur
        Task 1: b ← produce a subsequent block for B_i
        Task 2: B' ← notification from another worker
    end
    if Task 1 has been completed then
        append b to B_i
        notify workers in the network about B_i
    else if B' is longer than B_i then
        B_i ← B'
    endif
```

sending the complete sequence from root to end as part of such a message is an accurate, but also expensive approach, in terms of bandwidth, computation, and time. On the other hand, sending only the last block as part of the message is modest on resources, but can represent a communication conundrum whenever the worker being notified about a new block $x$ is not yet aware of the parent block of $x$. In contrast to slow systems, this situation may frequently occur in fast systems. The workaround is to use subsequent messages to query the previous blocks of $x$, as needed, thus extending the average duration of inter-working communication.

## 3 A Random Network Model for Blockchains

The network model generates a rooted tree representing a global blockchain from a collection of linked lists representing local blockchains (see Definition 2). It consists of three mechanisms, namely, growth, attachment, and broadcast. By growth it is meant that the number of blocks in the network increases by one at each time step. Attachment refers to the fact that new blocks connect to an existing block, while broadcast refers to the fact that the newly connected block is announced to the entire network. The model is parametric in a natural number $m$ specifying the number of workers, and two probability distributions $\alpha$ and $\beta$ governing the growth, attachment, and broadcast mechanisms. Internally, the growth mechanism creates a new block to be assigned at random among the $m$ workers by taking a sample from $\alpha$ (the time it takes to produce such a block) and broadcasts a synchronization message, whose reception time is sampled from $\beta$ (the time it takes the other workers to update their local blockchains with the new block).

A network at a given discrete step $n$ is represented as a rooted tree $T_n = (V_n, E_n)$, with nodes $V_n \subseteq \mathbb{N}$ and edges $E_n \subseteq V_n \times V_n$, and a map $w_n : \{0, 1, \ldots, m-1\} \to V_n$. A node $u \in V_n$ represents a block $u$ in the network and an edge $(u, v) \in E_n$ represents a directed edge from block $u$ to its *parent*

block $v$. The assignment $w_n(w)$ denotes the position (i.e., the last block in the local blockchain) of worker $w$ in $T_n$.

**Definition 3.** *(Growth model) Let $\alpha$ and $\beta$ be positive and non-negative probability distributions. The algorithm used in the network model starts with $V_0 = \{b_0\}$, $E_0 = \{\}$ and $w_0(w) = b_0$ for all workers $w$, being $b_0 = 0$ the root block (origin). At each step $n > 0$, $T_n$ evolves as follows:*

Growth. *A new block $b_n$ (or, simply, $n$) is created with production time $\alpha_n$ sampled from $\alpha$. That is, $V_n = V_{n-1} \cup \{n\}$.*

Attachment. *Uniformly at random, a worker $w \in \{0, 1, \ldots, m-1\}$ is chosen for the new block to extend its local blockchain. A new edge appears so that $E_n = E_{n-1} \cup \{(w_{n-1}(w), n)\}$, and $w_{n-1}$ is updated to form $w_n$ with the new assignment $w \mapsto n$, that is, $w_n(w) = n$ and $w_n(z) = w_{n-1}(z)$ for any $z \neq w$.*

Broadcast. *Worker $w$ broadcasts the extension of its local blockchain with the new block $n$ to any other worker $z$ with time $\beta_{n,z}$ sampled from $\beta$.*

The rooted tree generated by the model in Definition 3 begins with block 0 (the root) and adds new blocks $n = 1, 2, \ldots$ to some of the workers. At each step $n > 0$, a worker $w$ is selected at random and its local blockchain, $0 \leftarrow \cdots \leftarrow w_{n-1}(w)$, is extended to $0 \leftarrow \cdots \leftarrow w_{n-1}(w) \leftarrow n = w_n(w)$. This results in a concurrent random global behavior, inherent to distributed blockchain systems, not only because the workers are chosen randomly due to the proof-of-work scheme, but also because the communication delays bring some workers out of sync. It is important to note that the steps $n = 0, 1, 2, \ldots$ are logical time steps, not to be confused with the sort of time units sampled from the variables $\alpha$ and $\beta$. More precisely, although the model does not mention explicitly the time advancement, it assumes implicitly that workers are synchronized at the corresponding point in the logical future. For instance, if $w$ sends a synchronization message of a newly created block $n$ to another worker $z$, at the end of logical step $n$ and taking $\beta_{n,z}$ time, the message will be received by $z$ during the logical step $n' \geq n$ that satisfies $\sum_{i=n+1}^{n'} \alpha_i \leq \beta_{n,z} < \sum_{i=n+1}^{n'+1} \alpha_i$.

Another two reasonable assumptions are implicitly made in the model, namely: (i) the computational power of all workers is similar; and (ii) any broadcasting message includes enough information about the new and previous blocks, so that no re-transmission is required to fill block gaps (or, equivalently, that these re-transmission times are included in the delay sampled from $\beta$). Assumption (i) justifies why the worker producing the new block is chosen uniformly at random. Thus, instead of simulating the proof-of-work of the workers to know who will produce the next block and at what time, it is enough to select a worker uniformly and take a sample time from $\alpha$. Assumption (ii) helps in keeping the model description simple. Without Assumption (ii), it would be mandatory to explicitly define how to proceed when a worker is severely out of date and requires several messages to get synchronized.

In practice, the distribution $\alpha$ that governs the time it takes for the network, as a single entity, to produce a block is exponential with mean $\bar{\alpha}$. Since proof-of-work is based on finding a nonce that makes a hashing function fall into a specific set of targets, the process of producing a block is statistically equivalent to waiting for a success in a sequence of Bernoulli trials. Such waiting times would correspond –at first– to a discrete geometric distribution. However, because the time between trials is very small compared to the average time between successes (usually fractions of microseconds against several seconds or minutes), the discrete geometric distribution can be approximated by a continuous exponential distribution function. Finally, note that the choice of the distribution function $\beta$ that governs the communication delay, and whose mean is denoted by $\bar{\beta}$, heavily depends on the system under consideration and its communication details (e.g., its hardware and protocol).

## 4 Algorithmic Analysis of Blockchain Efficiency

This section presents an algorithmic approach to the analysis of blockchain efficiency. The algorithms are used to estimate the proportion of valid blocks that are produced during a fixed number of growth steps, based on the network model introduced in Section 3, for blockchains with fixed and unbounded number of workers. In general, although presented in this section for the specific purpose of measuring blockchain efficiency, these algorithms can be easily adapted to compute other metrics of interest, such as the speed of growth of the longest branch, the relation between confirmations of a block and the probability of being valid in the long term, or the average length of forks.

**Definition 4.** *Let $T_n = (V_n, E_n)$ be a blockchain that satisfies Definition 3. The* proportion of valid blocks *$p_n$ in $T_n$ is defined as the random variable:*

$$p_n = \frac{\max\{\text{dist}(0, u) \mid u \in V_n\}}{|V_n|}.$$

*The* proportion of valid blocks *$p$ produced for a blockchain (in the limit) is defined as the random variable:*

$$p = \lim_{n \to \infty} p_n.$$

*Their expected values are denoted with $\bar{p}_n$ and $\bar{p}$, respectively.*

Note that $\bar{p}_n$ and $\bar{p}$ are random variables particularly useful to determine some important properties of blockchains. For instance, the probability that a newly produced block becomes valid in the long run is $\bar{p}$. The average rate at which the longest branch grows is approximated by $\bar{p}/\bar{\alpha}$. Moreover, the rate at which invalid blocks are produced is approximately $(1 - \bar{p})/\bar{\alpha}$ and the expected time for a block to receive a confirmation is $\bar{\alpha}/\bar{p}$. Although $p_n$ and $p$ are random for any single simulation, their expected values $\bar{p}_n$ and $\bar{p}$ can be approximated by averaging several Monte Carlo simulations.

The three algorithms presented in the following subsections are sequential and single threaded[1], designed to compute the value of $p_n$ under the standard

[1] This would be mitigated by the fact that parallelization may be available for the Monte-Carlo simulations.

protocol (Algorithm 1). They can be used for computing $\bar{p}_n$ and, thus, for approximating $\bar{p}$ for large values of $n$. The first and second algorithms compute the *exact* value of $p_n$ for a bounded number of workers. While the first algorithm simulates the three mechanisms present in the network model (i.e., growth, attachment, and broadcast –see Definition 3), the second one takes a more time-efficient approach for computing $p_n$. The third algorithm is a fast approximation algorithm for $p_n$, useful in the context of an *unbounded* number of workers. It is of special interest for studying the efficiency of large and fast blockchain systems because its time complexity does not depend on the number of workers in the network.

### 4.1 Network Simulation with a Priority Queue

Algorithm 2 simulates the model with $m$ workers running concurrently under the standard protocol for up to $n$ logical steps. It uses a list $B$ of $m$ block sequences that reflect the local copy of each worker. The sequences are initially limited to the origin block 0 and can be randomly extended during the simulation. Each iteration of the main loop consists of four stages: (i) the wait for a new block to be produced, (ii) the reception of messages within a given waiting period, (iii) the addition of a block to the blockchain of a randomly selected worker, and (iv) the broadcasting of the new position of the selected worker in the shared blockchain to the other workers. The priority queue *pq* is used to queue messages for future delivery, thus simulating the communication delays. Messages have the form $(t', i, B')$, where $t'$ represents the arrival time of the message, $i$ is the recipient worker, and $B'$ the content that informs that a (non-specified) worker has the sequence of blocks $B'$. The statements $\alpha()$ and $\beta()$ draw samples from $\alpha$ and $\beta$, respectively.

The overall complexity of Algorithm 2 depends, as usual, on specific assumptions on its concrete implementation. First, let the time complexity to query $\alpha()$ and $\beta()$ be $O(1)$, which is a reasonable assumption in most computer programming languages. Second, note that the following time complexity estimates may be higher depending on their specific implementations (e.g., if a histogram is used instead of a continuous function for sampling these variables). In particular, consider two implementation variants. For both variants, the average length of the priority queue with arbitrarily large $n$ is expected to be $O(m)$, more precisely, $m\bar{\beta}/\bar{\alpha}$. Consider a scenario in which the statement $B_i \leftarrow B'$ is implemented by creating a copy in $O(n)$ time and the append statement is $O(1)$ time. The overall time complexity of the algorithm is $O(mn^2)$. Now consider a scenario in which $B_i \leftarrow B'$ merely copies the list reference in $O(1)$ time and the append statement creates a copy in $O(n)$ time. For the case where $n \gg m$, under the assumption that the priority queue has log-time insertion and removal, the time complexity is brought down to $O(n^2)$. In either case, the spatial complexity is $O(mn)$.

A key advantage of Algorithm 2 is that with a slight modification it can return the blockchain $s$ instead of the proportion $p_n$, which enables a richer analysis in the form of additional metrics different than $p$. For example, assume

**Algorithm 2:** Simulation of $m$ workers using a priority queue.

$t \leftarrow 0$<br>
$B \leftarrow [\,[0], [0], ..., [0]\,]$ *(m block sequences, 0 is the origin)*<br>
pq $\leftarrow$ empty priority queue<br>
**for** $k \leftarrow 1, ..., n-1$ **do**<br>
    $t \leftarrow t + \alpha()$<br>
    **for** $(t', i, B') \in$ pq with $t' < t$ **do** *(receive notifications)*<br>
        pop $(t', i, B')$ from pq<br>
        **if** $B'$ is longer than $B_i$ **then** $B_i \leftarrow B'$ **endif**<br>
    **end**<br>
    $j \leftarrow$ random_worker() *(block producer)*<br>
    append a new block $(k)$ to $B_j$<br>
    **for** $i \in \{0, ..., m-1\} \setminus \{j\}$ **do** *(publish notifications)*<br>
        push $(t + \beta(), i, B_j)$ to pq<br>
    **end**<br>
**end**<br>
$s \leftarrow \underset{s \in B}{\arg\max}\, |s|$ *(longest sequence)*<br>
**return** $|s|/n$

that $I$ denotes the random variable that describes the quantity of invalid blocks that are created between consecutive blocks. The expected value $E[I]$ can be estimated from $\bar{p}$ as $E[I] \approx (1 - \bar{p})/\bar{\alpha}$. Building a complete blockchain can be used to estimate not only $E[I]$, but also a complete histogram of $I$ and various properties it may possess.

### 4.2 A Faster Simulation Algorithm

**Algorithm 3:** Simulation of $m$ workers using a matrix $d$

$t_0, h_0, z_0 \leftarrow 0, 1, 0$<br>
$d_0 \leftarrow \langle 0, 0, ..., 0 \rangle$ *(m elements)*<br>
**for** $k \leftarrow 1, ..., n-1$ **do**<br>
    $j \leftarrow$ random_worker()<br>
    $t_k \leftarrow t_{k-1} + \alpha()$<br>
    $h_k \leftarrow 1 + \max\{h_i \,|\, i < k \wedge t_i + d_{i,j} < t_k\}$ (Algorithm 4)<br>
    $z_k \leftarrow \max(z_{k-1}, h_k)$<br>
    $d_k \leftarrow \langle \beta(), ..., \beta(), \overbrace{0}^{}, \beta(), ..., \beta() \rangle$ ($j$'th position)<br>
**end**<br>
**return** $z_{n-1}$

Algorithm 3 is a faster alternative to Algorithm 2. It uses a different encoding for the collection of local blockchains. In particular, Algorithm 3 stores the length of the blockchains instead of the sequences themselves. Thereby, it suppresses the need for a priority queue. Algorithm 4 offers an optimized routine that can be called from Algorithm 3.

**Algorithm 4:** Fast computation of $h_k$ given $t_i, z_i, h_i$ and $d_i$ for all $i < k$

$x, i \leftarrow 1, k-1$
**while** $i \geq 0$ and $x < z_i$ **do**
  **if** $t_i \leq t_k - d_{i,j}$ and $h_i > x$ **then**
    $x = h_i$
  **endif**
  $i \leftarrow i - 1$
**end**
**return** $1 + x$ *(compute* $h_k := 1 + \max\{h_i \mid i < k \wedge t_i + d_{i,j} < t_k\} \cup \{1\}$*)*

Let $t_k$ represent the (absolute) time at which block $k$ is created, $h_k$ the length of the local blockchain after being extended with block $k$, and $z_k$ the cumulative maximum given by

$$z_k := \max\{h_i \mid i \leq k\}.$$

The spatial complexity of Algorithm 3 is $O(mn)$ due to the computation of matrix $d$ and its time complexity is $O(nm + n^2)$ when Algorithm 4 is not used. Note that there are $n$ iterations, each requiring $O(n)$ and $O(m)$ time for computing $h_k$ and $d_k$, respectively. However, if Algorithm 4 is used for computing $h_k$, the average overall complexity is reduced. In the worst-case scenario, the complexity of Algorithm 4 is $O(k)$. However, the experimental evaluations suggest an average below $O(\bar{\beta}/\bar{\alpha})$ (constant with respect to $k$). Thus, the average runtime complexity of Algorithm 3 is bounded by $O\left(nm + \min\{n^2, n + n\bar{\beta}/\bar{\alpha}\}\right)$, and this corresponds to $O(nm)$, unless the blockchain system is extremely fast ($\bar{\beta} \gg \bar{\alpha}$).

### 4.3 An Approximation Algorithm for Unbounded Number of Workers

Algorithms 2 and 3 compute the value of $p_n$ for a *fixed* number $m$ of workers. Both algorithms can be used to compute $p_n$ for different values of $m$. However, the time complexity of these two algorithms heavily depends on the value of $m$, which presents a practical limitation when faced with the task of analyzing large blockchain systems. This section introduces an algorithm for approximating $p_n$ for an unbounded number of workers. It also presents formal observations that support the proposed approximation.

Recall that $p_n$ can be used as a measure of efficiency in terms of the proportion of valid blocks that have been produced up to step $n$ in the blockchain

$T_n = (V_n, E_n)$. Formally:

$$p_n = \frac{\max\{\text{dist}(0, u) \mid u \in V_n\}}{|V_n|}.$$

This definition assumes a fixed number of workers. That is, $p_n$ can be written as $p_{m,n}$ to represent the proportion of valid blocks in $T_n$ *with* $m$ workers. For the analysis of large blockchains, the challenge is to find an efficient way to estimate $p_{m,n}$ for large values of $m$ and $n$. In other words, to find an efficient algorithm for approximating the random variables $p_n^*$ and $p^*$ defined as:

$$p_n^* = \lim_{m\to\infty} p_{m,n} \qquad \text{and} \qquad p^* = \lim_{n\to\infty} p_n^* = \lim_{m,n\to\infty} p_{m,n}.$$

The proposed approach modifies Algorithm 3 by suppressing the matrix $d$. The idea is to replace the need for computing $d_{i,j}$ by an approximation based on the random variable $\beta$ and the length of the blockchain $h_k$ in each iteration of the main loop. Note that the first row can be assumed to be 0 wherever it appears because $d_{0,j} = 0$ for all $j$. For the remaining rows, an approximation is introduced by observing that if an element $X_m$ is chosen at random from the matrix $d$ of size $(n-1)\times m$ (i.e., matrix $d$ without the first row), then the cumulative distribution function of $X_m$ is given by

$$P(X_m \le r) = \begin{cases} 0 & , \ r < 0 \\ \frac{1}{m} + \frac{m-1}{m} P(\beta() \le r) & , \ r \ge 0, \end{cases}$$

where $\beta()$ is a sample from $\beta$. This is because the elements $X_m$ of $d$ are either samples from $\beta$, whose domain is $\mathbb{R}_{\ge 0}$, or 0 with a probability of $1/m$ since there is one zero per row. Therefore, given that the following functional limit converges uniformly (see Theorem 1 below),

$$\lim_{m\to\infty} \left( r \overset{f_m}{\mapsto} P(X_m \le r) \right) = \left( r \overset{f}{\mapsto} P(\beta() \le r) \right),$$

each $d_{i,j}$ can be approximated by directly sampling the distribution $\beta$. As a result, Algorithm 4 can be used for computing $h_k$ by replacing $d_{i,j}$ with $\beta()$.

**Theorem 1.** *Let* $f_k(r) := P(X_k \le r)$ *and* $g(r) := P(\beta() \le r)$. *The functional sequence* $\{f_k\}_{k=1}^{\infty}$ *converges uniformly to* $g$.

*Proof.* Let $\epsilon > 0$. Define $n := \left\lceil \frac{1}{2\epsilon} \right\rceil$ and let $k$ be any integer $k > n$. Then

$$\begin{aligned} \sup |f_k - g| &= \sup \left\{ \left| \frac{1}{k} + \left( \frac{k-1}{k} - 1 \right) P(\beta() \le r) \right| : r \ge 0 \right\} \\ &\le \frac{1}{k} + \frac{1}{k} \sup \{P(\beta() \le r) : r \ge 0\} \\ &= \frac{1}{k} + \frac{1}{k} \\ &< \frac{2}{n} \le \epsilon. \end{aligned}$$

□

Using Theorem 1, the need for the bookkeeping matrix $d$ and the selection of a random worker $j$ are discarded from Algorithm 3, resulting in Algorithm 5. The proposed algorithm computes $p_n^*$, an approximation of $\lim_{m\to\infty} p_{m,n}$ in which the matrix entries $d_{i,j}$ are replaced by samples from $\beta$, each time they are needed, thus ignoring the arguably negligible hysteresis effects.

**Algorithm 5:** Approximation for $\lim_{m\to\infty} p_{m,n}$ simulation

$t_0, h_0, z_0 \leftarrow 0, 0, 0$
**for** $k \leftarrow 1, ..., n-1$ **do**
  $t_k \leftarrow t_{k-1} + \alpha()$
  $h_k \leftarrow 1 + \max\{h_i \mid i < k \wedge t_i + \beta() < t_k\} \cup \{1\}$ (Algorithm 4*)
  $z_k \leftarrow \max(z_{k-1}, h_k)$
**end**
**return** $z_{n-1}$
Algorithm 4* stands for Algorithm 4 with $\beta()$ instead of $d_{i,j}$ (approximation)

The time complexity of Algorithm 5 implemented by using Algorithm 4 with $\beta()$ instead of $d_{i,j}$ is $O(n^2)$ and its space complexity is $O(n)$. If the pruning algorithm is used, the time complexity drops below $O(n + n\bar{\beta}/\bar{\alpha}))$ according to experimentation. This complexity can be considered $O(n)$ as long as $\bar{\beta} \not\gg \bar{\alpha}$.

## 5 Empirical Evaluation of Blockchain Efficiency

This section presents an experimental evaluation of blockchain efficiency in terms of the proportion of valid blocks produced by the workers for the global blockchain. The model in Section 3 is used as the mathematical framework, while the algorithms in Section 4 are used for experimental evaluation on that framework. The main claim is that, under certain conditions, the efficiency of a blockchain can be expressed as a ratio between $\bar{\alpha}$ and $\bar{\beta}$. Experimental evaluations provide evidence on why Algorithm 5 –the approximation algorithm for computing the proportion of valid blocks in a blockchain system with an unbounded number of workers– is an accurate tool for computing the measure of efficiency $p^*$.

Note that the speed of a blockchain can be characterized by the relationship between the expected values of $\alpha$ and $\beta$.

**Definition 5.** *Let $\alpha$ and $\beta$ be the distributions according to Definition 3. A blockchain is classified as:*

- slow *if* $\bar{\alpha} \gg \bar{\beta}$*,*
- chaotic *if* $\bar{\alpha} \ll \bar{\beta}$*, and*
- fast *if* $\bar{\alpha} \approx \bar{\beta}$*.*

Definition 5 captures the intuition about the behavior of a global blockchain in terms of how alike are the times required for producing a block and for local block synchronization. Note that the Bitcoin implementation is classified as a slow blockchain system because the time between the creation of two consecutive blocks is much larger than the time it takes for local blockchains to synchronize. In chaotic blockchains, a dwarfing synchronization time means that basically no (or relatively little) synchronization is possible, resulting in a blockchain in which rarely any block would be part of "the" valid chain of blocks. A fast blockchain, however, is one in which both the times for producing a block and broadcasting a message are similar. The two-fold goal of this section is first, to analyze the behavior of $\bar{p}^*$ for the three classes of blockchains, and second, to understand how the trade-off between production speed and communication time affects the efficiency of the data structure by means of a formula.

In favor of readability, the experiments presented next identify algorithms 3 and 5 as $A_m$ and $A_\infty$, respectively. Furthermore, the claims and experiments assume that the distribution $\alpha$ is exponential, which holds true for proof-of-work systems.

**Claim 1** *Unless the system is chaotic, the hysteresis effect of the matrix entries $d_{i,j}$ in $A_m$ is negligible. Moreover,* $\lim_{m\to\infty} A_m(n) = A_\infty(n)$.

Note that Theorem 1 implies that if the hysteresis effect of the random variables $d_{i,j}$ is negligible, then Algorithm 5 is a good enough approximation of Algorithm 3. However, it does not prove that this assertion holds in general. Experimental evaluation suggests that this is indeed the case, as stated in Claim 1.

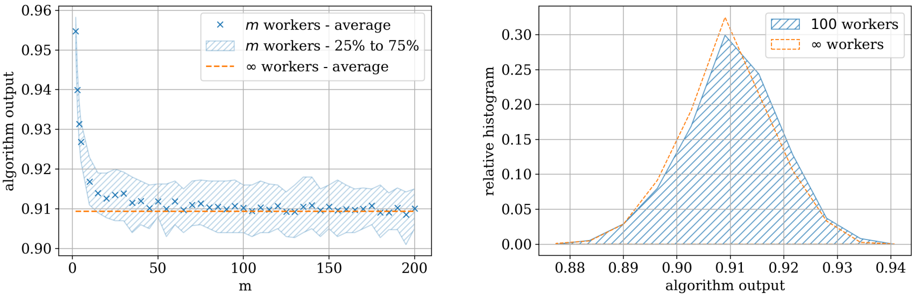

(a) Evolution of $A_m$ to $A_\infty$ as $m$ grows. Simulation runs contain at least 100 samples per point.

(b) High similarity between the p.d.f. of $A_{100}$ and $A_\infty$. Simulation runs contain at least 1000 samples in total.

Fig. 3: Algorithmic simulation of $n = 1000$ blocks with $\bar{\alpha} = 1$, $\bar{\beta} = 0.1$, and $\beta$ exponential. The number of samples and the size of the blockchain $n$ are chosen such that the execution time on a standard cpu lies below a few seconds.

Figure 3 summarizes the average output of $A_m$ and the region that contains half of these outputs, for several values of $m$. All outputs seem to approach that of $A_\infty$, not only for the expected value (Figure 3.(a)), but also in terms of the generated p.d.f. (Figure 3.(b)). Similar results were obtained with several distribution functions for $\beta$. In particular, the exponential, chi-squared, and gamma probability distribution functions were used (with $k \in \{1, 1.5, 2, 3, 5, 10\}$), all with different mean values. The resulting plots are similar to the ones depicted in Figure 3.

As the quotient $\bar{\beta}/\bar{\alpha}$ grows beyond 1, the convergence of $A_m$ becomes much slower and the approximation error is noticeable. An example is depicted in Figure 4, where a blockchain system produces on average 10 blocks during the transmission of a synchronization message (i.e., the system is classified as chaotic). Even after considering 1000 workers, the shape of the p.d.f. is shifted considerably. The error can be due to: (i) the hysteresis effect that is ignored by $A_\infty$; or (ii) the slow rate of convergence. In any case, the output of this class of systems is very low, making them unstable and useless in practice.

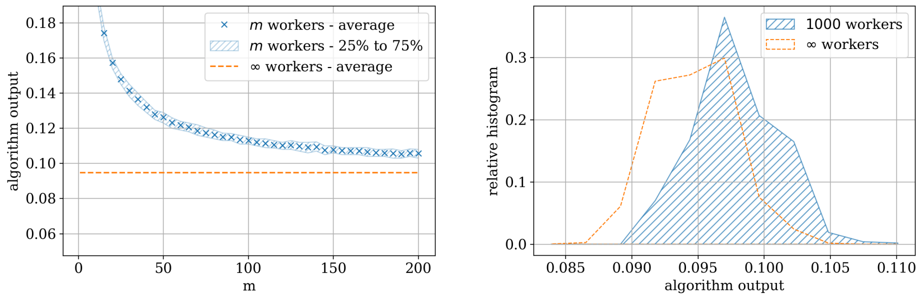

Fig. 4: For chaotic systems, the convergence is slow and the approximation error is large: with 1000 workers there is still an average output shift of around 0.005.

An intuitive conclusion about blockchain efficiency and speed of block production is that slower systems tend to be more efficient than faster ones. That is, faster blockchain systems have a tendency to overproduce blocks that will not be valid.

**Claim 2** *If the system is either slow or fast, then*

$$\bar{p}^* = \frac{\bar{\alpha}}{\bar{\alpha} + \bar{\beta}}.$$

Figure 5 presents an experimental evaluation of the proportion of valid blocks in a blockchain in terms of the ratio $\bar{\beta}/\bar{\alpha}$. For the left and right plots, the horizontal axis represents how fast blocks are produced in comparison with how slow synchronization is achieved. If the system is slow, then efficiency is high because most newly produced blocks tend to be valid. If the system is fast, however, then efficiency is balanced because the newly produced blocks are likely to either become valid or invalid with equal likelihood. Finally, note that for fast and chaotic blockchains, say for $10^{-1} \leq \bar{\beta}/\bar{\alpha}$, there is still a region in which efficiency is arguably high. As a matter of fact, even if synchronization of local blockchains takes on average a tenth of the time it takes to produce a block, in general, the proportion of blocks that become valid is almost 90%. In practice, this observation can bridge the gap between the current use of blockchains as slow systems and the need for faster blockchains.

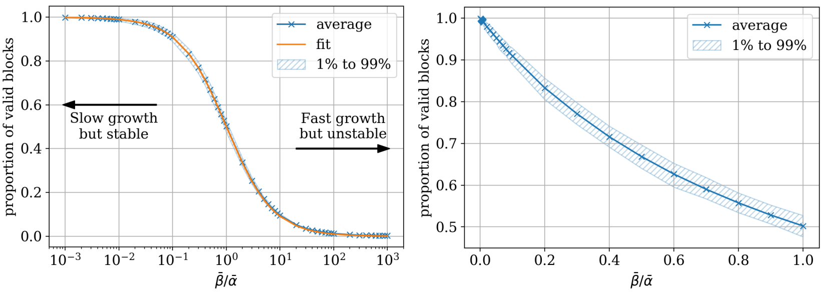

Fig. 5: Effect of speed on the proportion of valid blocks.

## 6 Related Work and Concluding Remarks

A comprehensive account of the vast literature on complex networks is beyond the scope of this work. The aim here is more modest, namely, the focus is on related work proposing and using formal and semi-formal algorithmic approaches to evaluate properties of blockchain systems. There are a number of recent studies that focus on the analysis of blockchain properties with respect to meta-parameters. Some of them are based on network and node simulators. Other studies conceptualize different metrics and models that aim to reduce the analysis to the essential parts of the system.

In [10], A. Gervais et al. introduce a quantitative framework to analyze the security and performance implications of various consensus and network parameters of proof-of-work blockchains. They devise optimal adversarial strategies for several attack scenarios while taking into account network propagation. Ultimately, their approach can be used to compare the tradeoffs between blockchain performance and its security provisions. Y. Aoki et al. [2] propose SimBlock, a blockchain network simulator in which blocks, nodes, and the network itself can be instantiated by using a comprehensive collection of parameters, including the propagation delay between nodes. Towards a similar goal, J. Kreku et al. [19] show how to use the Absolut simulation tool [28] for prototyping blockchains in different environments and finding optimal performance, given some parameters, in constrained platforms such as Raspberry Pi and Nvidia Jetson Tk1.

R. Zhang and B. Preneel [29] introduce a multi-metric evaluation framework to quantitatively analyze proof-of-work protocols. Their systemic security analysis in seven of the most representative and influential alternative blockchain designs concludes that none of them outperforms the so-called Nakamoto Consensus in terms of either the chain quality or attack resistance. All these efforts have in common that simulation-based analysis is used to understand non-functional requirements of blockchain designs such as performance and security, up to a high degree of confidence. However, in most of the cases the concluding results are tied to a specific implementation of the blockchain architecture. The model and algorithms presented in this work can be used to analyze each of these scenarios in a more abstract fashion by using appropriate parameters for simulating the blockchain growth and synchronization.

An alternative approach for studying blockchains is through formal semantics. G. Rosu [24] takes a novel approach to the analysis of blockchain systems by focusing on the formal design, implementation, and verification of blockchain languages and virtual machines. His approach uses continuation-based formal semantics to later analyze reachability properties of the blockchain evolution with different degrees of abstraction. In this direction of research, E. Hildenbrandt et al. [14] present KEVM, an executable formal specification of Ethereum's virtual machine that can be used for rapid prototyping, as well as a formal interpreter of Ethereum's programming languages. C. Kaligotla and C. Macal [17] present an agent-based model of a blockchain systems in which the behavior and decisions made by agents are detailed. They are able to implement a generalized simulation and a measure of blockchain efficiency from an agent choice and energy cost perspective. Finally, J. Göbel et al. [11] use Markov models to establish that some attack strategies, such as selfish-mine, causes the rate of production of orphan blocks to increase. The research presented in this manuscript uses random networks to model the behavior of blockchain systems. As future work, the proposed model and algorithms can be specified in a rewrite-based framework such as rewriting logic [20], so that the rule-based approach in [24,14] and the agent-based approach in [17] can both be extended to the automatic analysis of (probabilistic) temporal properties of blockchains. Moreover, as it is usual in a random network approach, topological properties of blockchain systems can be studied with the help of the model proposed in this manuscript.

In general, this paper differs from the above studies in the following aspects. The proposed analysis is not based on an explicit low-level simulation of a network or protocol; it does not explore the behavior of blockchain systems under the presence attackers. Instead, this work simulates the behavior of blockchain efficiency from a meta-level perspective and investigates the strength of the system with respect to shortcomings inherent in its design. Therefore, the proposed analysis differs from [10,2,19,29] and is rather closely related to studies which consider the core properties of blockchain systems prior to attacks [17,29]. The bounds for the meta-parameters are more conservative and less secure, compared to scenarios in which the presence of attackers is taken into account. Finally, with respect to studying blockchains through formal semantics, the proposed analysis is able to consider an artificial but convenient scenario of having an infinite number of concurrent workers. Formal semantics, as well as other related simulation tools, cannot currently handle such scenarios.

This paper presented a network model for blockchains and showed how the proposed simulation algorithms can be used to analyze the efficiency (in terms of production of valid blocks) of blockchain systems. The model is parametric on: (i) the number of workers (or nodes); and (ii) two probability distributions governing the time it takes to produce a new block and the time it takes the workers to synchronize their local copies of the blockchain. The simulation algorithms are probabilistic in nature and can be used to compute the expected value of several metrics of interest, both for a fixed and unbounded number of workers, via Monte Carlo simulations. It is proven, under reasonable assumptions, that the fast approximation algorithm for an unbounded number of workers yields accurate estimates in relation to the other two exact (but much slower) algorithms. Claims –supported by extensive experimentation– have been proposed, including a formula to measure the proportion of valid blocks produced in a blockchain in terms of the two probability distributions of the model. The model, algorithms, and experiments provide insights and useful mathematical tools for specifying, simulating, and analyzing the design of fast blockchain systems in the years to come.

Future work on the analytic analysis of the experimental observations contributed in this work should be pursued. This includes proving the two claims in Section 5. First, that hysteresis effects are negligible unless the system is extremely fast. Second, that the expected proportion of valid blocks in a blockchain system is given by $\bar{\alpha}/(\bar{\alpha}+\bar{\beta})$, being $\bar{\alpha}$ and $\bar{\beta}$ the mean of the probability distributions governing block production and communication times, respectively. Furthermore, the generalization of the claims to non-proof-of-work schemes, i.e. to different probability distribution functions for specifying the time it takes to produce a new block may also be considered. Finally, the study of different forms of attack on blockchain systems can be pursued with the help of the proposed model.

*Acknowledgments.* This research was supported by the Center of Excellence and Appropriation in Big Data and Data Analytics (CAOBA), founded by the Ministry of Information Technologies and Telecommunications of Colombia (MinTIC) and the Colombian Administrative Department of Science, Technology and Innovation (COLCIENCIAS) under grant no. FP44842-anex46-2015.
